# Postpartum hepatitis and host immunity in pregnant women with chronic HBV infection

**DOI:** 10.3389/fimmu.2022.1112234

**Published:** 2023-01-04

**Authors:** Lu Zhang, Tingting Jiang, Ying Yang, Wen Deng, Huihui Lu, Shiyu Wang, Ruyu Liu, Min Chang, Shuling Wu, Yuanjiao Gao, Hongxiao Hao, Ge Shen, Mengjiao Xu, Xiaoxue Chen, Leiping Hu, Liu Yang, Xiaoyue Bi, Yanjie Lin, Yao Lu, Yuyong Jiang, Minghui Li, Yao Xie

**Affiliations:** ^1^ Department of Hepatology Division 2, Beijing Ditan Hospital, Capital Medical University, Beijing, China; ^2^ Hepatology Department 2, Xingtai Second Hospital, Xingtai, China; ^3^ Department of Obstetrics and Gynecology, Wuhan Children’s Hospital (Wuhan Maternal and Child Healthcare Hospital), Tongji Medical College, Huazhong University of Science and Technology, Wuhan, China; ^4^ Department of Hepatology Division 2, Peking University Ditan Teaching Hospital, Beijing, China; ^5^ Center of Integrative Medicine, Beijing Ditan Hospital, Capital Medical University, Beijing, China

**Keywords:** pregnant, HBV, immune tolerant, treg, postpartum hepatitis, antiviral therapy (AVT)

## Abstract

In order to develop immune tolerant to the fetal, maternal immune system will have some modification comparing to the time before pregnancy. Immune tolerance starts and develops at the maternal placental interface. In innate immunity, decidual natural killer (dNK) cells, macrophages and dendritic cells play a key role in immue tolerance. In adaptive immunity, a moderate increase of number and immune inhibition function of regulatory T cells (Treg) are essential for immune tolerance. The trophoblast cells and immune cells expressing indoleamine 2,3-dioxygenase (IDO), the trophoblast cells expressing HLA-G, and Th1/Th2 shifting to Th2 dominant and Th17/Treg shifting to Treg domiant are in favor of maternal fetal immune tolerance. Steroids (estrogen and progesterone) and human chorionic gonadotropin (HCG) also participate in immune tolerance by inducing Treg cells or upregulating immunosuppressive cytokines. Most of the patients with chronic HBV infection are in the “HBV immune tolerance period” before pregnancy, and the liver disease is relatively stable during pregnancy. In chronic HBV infection women, after delivery, the relative immunosuppression *in vivo* is reversed, and Th1 is dominant in Th1/Th2 and Th17 is dominant in Th17/Treg balance. After delivery, the number of Treg decrease and NK cells increase in quantity and cytotoxicity in peripheral blood. Liver NK cells may cause liver inflammation through a non-antigen specific mechanism. After delivery, the number of CD8^+^ T cells will increase and HBV specific T cell response recovers from the disfunction in pregnancy. Under the background of postpartum inflammation, the rapid decrease of cortisol after delivery, and especially the enhancement of HBV specific T cell response induced by HBV DNA and cytokines, are the main reasons for postpartum hepatitis. HBeAg positive, especially HBeAg<700 S/CO, and HBV DNA>3-5Log_10_IU/ml are risk factors for postpartum hepatitis. Antiviral treatment in late pregnancy can reduce the incidence of mother to child transmission (MTCT) in chronic HBV infection women. Chronic HBV infection women have hepatitis both during pregnancy and more often in 12 weeks postpartum. It is generally agreed that postpartum hepatitis is mild symptoms and self-limited. Delaying drug withdrawal to 48 weeks can increase the seroconversion rate of HBeAg in delivery women with elevated alanine aminotransferase (ALT) in pregnancy.

In pregnant women with chronic HBV infection, a certain proportion of postpartum hepatitis may occur after delivery. The mechanisms of postpartum hepatitis are complex, involving the changes in the immune system and pregnancy-associated hormone of women in pregnancy and after delivery, the natural stage of chronic HBV infection period, whether preventive antiviral therapy (AVT) and the time of withdrawal AVT after delivery.

## Definition and characteristics of postpartum hepatitis

### Definition of postpartum hepatitis

Postpartum hepatitis refers to the occurrence of hepatitis after delivery, which is characterized by the elevated ALT with or without fatigue and corresponding digestive tract symptoms. The standards of ALT elevation vary from>1 upper limit of normal range (ULN) or >2ULN to >5ULN. Different countries have different ULN standards, most of which are >40U/L, but >25U/L or >19U/L for female in the United States (1). At present, research on postpartum hepatitis has focused on women with chronic HBV infection.

### Characteristic of postpartum hepatitis

Postpartum hepatitis mostly occurs within 12 weeks after delivery, but a few also occur in 12-24 weeks. The two peak periods of postpartum hepatitis are 3-4 weeks and 9-12 weeks respectively (2). If postpartum follow-up is only 12 weeks, postpartum hepatitis occurring after 12 weeks is often not monitored. The degree of postpartum hepatitis is mostly asymptomatic or mild to moderate. There is little chance of elevated bilirubin level or clinical decompensation of liver function. Postpartum hepatitis, either without oral AVT in late pregnancy or withdrawal AVT after delivery, is usually self-limited and does not require medical intervention (2–4). It is occasionally reported that a few patients have severe postpartum hepatitis flare (5). In chronic HBV infection women, the incidence of hepatitis during pregnancy can reach 6-14%, and it is more common after delivery (5). In chronic HBV infection women, percentage of postpartum hepatitis is 3.5%-50% (2, 4). The use of oral AVT to prevent MTCT in late pregnancy can also reduce the incidence of postpartum hepatitis. Postpartum hepatitis associated with MTCT is usually accompanied by a mild flare, usually without the need for AVT again (3).

## Changes of host immune system in pregnancy

### Innate and Adaptive Immune Responses in Pregnancy

The maternal-fetal interface mainly consists of trophoblast cells from the embryo and decidua from the pregnant women. In order to adapt to those process, the uterus innate immune system is activated in the early pregnancy, then gradually weakens in most of the pregnancy, and reactivates in the late pregnancy (6). The enhancement of innate immunity at the early pregnancy plays an important action in trophoblast growth, angiogenesis and placentation. The maternal placenta is rich in white blood cells, including about 70% decidual natural killer cells (dNK), 20% mononuclear macrophages, and roughly 10% T cells (7).

#### Changes of innate immunity during pregnancy

NK cells come from bone marrow and circulate to decidual tissue of uterus. During pregnancy, the quantity, phenotype and function of dNK cells at the maternal-fetal interface are different from those before pregnancy. The percentage of dNK cells increased in the first trimester of pregnancy accounting for 70-90% of decidual mononuclear cells, then decreased gradually in the second and third trimesters of pregnancy accounting for 35-40% (8, 9). The function of dNK cells may be influenced by the hormone level during pregnancy (10). High levels of adrenocortical hormone (ACH) during pregnancy directly inhibit NK cell activity (10). The phenotype of dNK cells is different from that of peripheral blood (11, 12). It is CD56^bright^CD16^-^NK, which can recognize non classical MHC-I molecules, and has weak cytotoxicity (8, 10). dNK cells can produce generous TNF-α and IFN-α which promote the invasion of extravillous trophoblast (EVT) cells and the formation of decidual vessels and spiral arteries (8, 9, 11). dNK cell function is regulated by the balance of inhibitory and active receptor signals (6). During pregnancy, the expression of dNK activated receptor NKG2D decreased, and the expression of inhibitory receptor CD94/NKG2A increased.The basic feature of inhibitory receptor is the structure of immunoreceptor tyrosine inhibitory motif (ITIM) in the cytoplasm. KIR can transmit inhibition signal through ITIM, thus inhibiting NK cells’ killing activity against target cells. The dysfunction of dNK is related to abnormal pregnancy, such as recurrent abortion and fetal growth retardation.

In pregnancy, the number of circulating monocytes and granulocytes increased and they were activated by up-regulating activation markers of CD11b and CD14, but the phagocytosis of monocytes decreased (13, 14). The decidual MФs mainly remodel the uterine blood vessels, participating in the regulation of immune tolerance and antigen presentation (13, 15). In early pregnancy, MФs actively remove apoptotic cells, produce proteases that degrade extracellular matrix, and participate in uterine vascular remodeling (16). MФ has high plasticity and can polarize into different phenotypes under the stimulation of different microenvironments. Decidual MФs are classified into classically activated macrophages (M1) and alternatively activated macrophages (M2) (16). M1 is dominant during both embryo implantation and during labor. MФs in decidua can induce Foxp3^+^Treg proliferation *in vitro*. Most decidual MФs produce indoleamine 2,3-dioxygenase (IDO), which is conducive to maternal immune tolerance to the half allogeneic fetus. In early pregnancy, most decidual T cells show strong expression of PD-1. Decidual MФs express B7 family costimulatory molecules and then inhibit T cells from producing IFN-γ through B7-H1/PD-1 signal pathway (17).

Dendritic cells (DC), derived from bone marrow hematopoietic stem cells, are the most important professional antigen presenting cells in the body. DC also acts as an important immune cell for immune tolerance in pregnancy. DC is divided into mature DC and immature DC. IDC is the main type of DC, and only a few mature CD83^+^DC in endometrium during pregnancy. IDC has strong antigen capture function, but weak antigen presentation function (18). Without the costimulatory signal on the surface, iDC can induce the disability of effector T lymphocytes (Teff), induce the production of Tregs, and promote immune tolerance in maternal fetal interface. IDC induces antigen specific T cell tolerance by secretes IL-10 and TGF-β. In the mouse model, DC at the maternal and fetal interface is difficult to migrate to the lymphatic vessels of the uterus and lymph nodes even if stimulated by LPS, which can minimize the exposure of fetal or placental antigens to immunogenic T cells and is beneficial to maternal fetal immune tolerance (19).

Other immune cells, such as mast cells. Mast cells were stimulated by cell surface-expressed pattern recognition receptors (PRR) from bacteria, released IL-6, leukotrienes, TNF and histamine. Two receptors of H2R and H4R in mast cells regulate the production of Treg (20, 21). The number of neutrophils from maternal fetal interfaces remained relatively constant during pregnancy and began to increase rapidly before delivery. In the first-trimester, the maternal leukocytes of decidua have significant function in the implantation and early development of embryo and placenta. Neutrophils produce reactive oxygen and lytic enzymes by phagocytosis of bacteria, and promote inflammatory cascade reaction (22).

#### Changes of adaptive immunity during pregnancy

The absolute counts of T, B lymphocytes and CD4^+^, CD8^+^ T cell subsets in early pregnancy decreased, and the proportion of lymphocytes remained unchanged (9). T cells account for 10%~15% of white blood cells in decidual tissue in early pregnancy, and this proportion increases to 70% in late pregnancy (7).

Treg cells are an important CD4^+^ T lymphocyte subset of immune tolerance in pregnancy (23–25). Treg cells are distributed in iliac and inguinal lymph nodes, spleen and peripheral blood, and later found in uterus (26). The exhaustion of Tregs will lead to pathological pregnancy. At present, the research mainly focuses on the inducing factors of amplification and the molecular action mechanism of Tregs. During early pregnancy, the number of Tregs in the pregnant woman increased and migrated to the surface of the mother and fetus (27). During the third trimester of pregnancy and after delivery, the number of Treg cells decreased significantly (28). CCL22/CCL17 at the maternal fetal interface can chemotactic Treg cells. Treg surface molecules include Foxp3, GITR (glucocorticoid induced TNF receptor), CTLA-4, and TGF-β. Tregs are usually represented by CD4^+^CD25^+^FoxP3^+^Tregs (24, 26). TGF-β can regulate the differentiation of Th17 cells and Treg cell lineage. The interaction between GITR and its ligand can eliminate the inhibition of CD25^+^CD4^+^Tregs and enhance the activation, proliferation and cytokine production of T cells. Treg exerts its immune regulation function mainly through two ways: 1. Direct contact between cells. Treg can inhibit the proliferation of immune cells by directly binding with corresponding receptors of CD4^+^ T, CD8^+^ T and DCs through surface molecules (25, 29). For example, the interaction between the Treg inhibitory receptor CTLA-4 and its ligand CD80/CD86 induces macrophages or DCs to express indolamine 2,3-dioxygenase, which inhibits T cell activity and promotes maternal tolerance to the fetus (30, 31). Treg can inhibit the activation and amplification of immune cells such as CD4^+^ T and CD8^+^ T cells. 2. Secretion of inhibitory cytokines. For example, Tregs secretes IL-10 and TGF-β which are also important for self reproduction. In normal pregnancy, Tregs promote Th2 immune tolerance, but do not reduce Th1 cytokines, promoting maternal fetal immune tolerance (28, 32). The interaction between dNK and monocytes can also activate Treg (33). Many cytokines and hormones can regulate Treg production. During the first six months of pregnancy, semen contains TGF-β, IL-10 and prostaglandin E2 (PGE2), paternal antigen, can significantly increase Treg cells in peripheral blood and decidual tissue (34). The number of Treg cells increased significantly with the dramatic increase of human chorionic gonadotropin (HCG) (35).

The most abundant lymphocytes in decidual tissue are CD8^+^ T cells. Exhausted and senescent CD8^+^ T cells exist at the maternal fetal interface (36). In the maternal fetal interface, the inhibition receptors of CD8^+^ T cells such as CTLA4, PD1 and LAG3, suggest a phenotype of T cell depletion or dysfunction. The function of CD8^+^ T cells is temporarily inhibited and can still proliferate and produce IFN-γ, TNF-α, perforin and granzyme when stimulated (37, 38). During early pregnancy, the regulatory CD8^+^T cell population co-expressing PD-1 and T cell immunoglobulin and mucin domain containing protein 3 (Tim-3) which are enriched in decidua, and can proliferate and produce Th2 type cytokines (39). In the first trimester of pregnancy, apoptosis of T cells which express Fas on the surfaces of cells were induced by FasL from maternal decidua and placental trophoblast cells (40). Antigen presenting cells (APC) which express PD-L1 combined with T cells which express PD1 to induce T cell apoptosis (41). αβT cells are the most important T cells in the body. Due to the lack of effective stimulus of MHC molecules, the harm of αβT cells to the fetus are almost inhibited in pregnant women. TCRγδT cells involved in the immune tolerance of pregnancy (25, 42). By recognizing HLA G and HLA E antigens of trophoblasts, r&T cells are activated and release inhibitory cytokines of IL-10, TGF-β, and inhibit their cytotoxicity in pregnancy (6).

B lymphocyte cells have the ability to produce specific antibodies, in addition, they have the function of secondary antigen presenting cells, and can produce various immune regulatory cytokines. Adaptive cellular immunity was down regulated and humoral immunity was up regulated during pregnancy (43). Regulatory B cells (Bregs) are B lymphocyte subsets with strong immunosuppressive function. Studies have shown that under physiological conditions, the number of Breg is very small. However, in animal experiments, it is found that mature B cells of pregnant rats gather in the abdominal cavity and uterine lymph nodes (44), which may increase the number of serum immunoglobulins (43). Breg inhibits the toxic activity of Th1, Th17, and CD8^+^ T cells, and they can also induce CD4^+^ T cells to differentiate into FoxP3^+^Tregs (45, 46). Breg also inhibited TNF-α production by monocytes and IFN-α production by plasmacytoid dendritic cells (PDCs). Bregs are negative regulators of the body’s immune system through the anti-inflammatory cytokines IL-10, TGF-β. Breg inhibits the immunity through direct contact between cells or the indirect action of cytokines such as IL-35 (45).

Myeloid-derived suppressor cells (MDSCs) are a group of heterogeneous immature cells from bone marrow. MDSCs have immunosuppression function, and become new immune regulating cells at the maternal-fetal interface. MDSCs play an inhibitory role in normal pregnancy. MDSCs are characterized by myeloid origin and immature state, which can inhibit T cells. MDSCs suppress immunity through two ways. One way is through arginase 1 (Arg1), iDO, NO, ROS, and PGE2. Arg1, causing hunger environment by consuming essential amino acids such as arginine and tryptophan, leads to T cell inhibition (47). Another way is through TGF-β which can induce Treg production (48). MDSCs can inhibit NK through NO, ROS and inhibitory receptor TIGIT. MDSCs can also inhibit the activation of T cells by DCs (49).

Natural killer T (NKT) cell is a special T cell subgroup both with T cell receptor and NK cell receptor on the cell. NKT cells can produce a large number of cytokines, and play a similar cytotoxic role to NK cells. The NKT activation process requires the mutual activation of APC and NKT. The decidual NKT has obvious Th2 tendency, which is very important at the maternal fetal interface (50). Under normal conditions, decidual NKT inhibits immune rejection against paternal antigens, which is beneficial to the growth of trophoblast.

### Mechanism of immune tolerance to “half allogeneic” fetus during pregnancy

**Figure 1:**
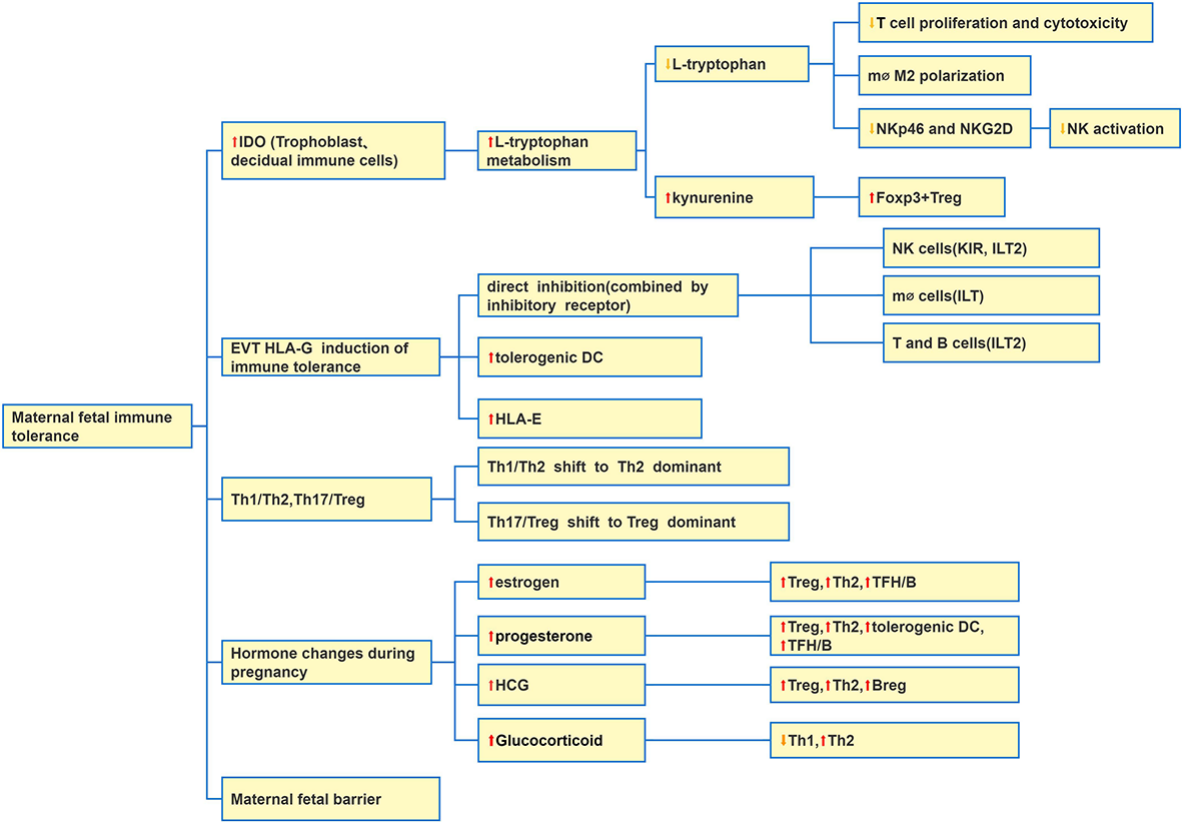
Causes of maternal fetal immune tolerance. There are five main reasons: IDO at the maternal-fetal interface increases which induces maternal fetal immune tolerance; EVTs express HLA-G to induce immune tolerance; Changes of Th1, Th2, Th17 and Treg balance; hormone change during pregnancy; maternal-fetal barrier. Abbreviation: Extraxtravillous Trophoblast (EVT).

There are a large number of immune cells infiltrating in the uterus which construct the endometrial immune microenvironment and enable the embryo to obtain “ immune privilege” (51, 52). At present, it is generally believed that in addition to the placental barrier, maternal fetal immune tolerance maintains mainly by increasing the expression of human leukocyte antigen (HLA) G molecule in the placenta, Th1/Th2 and T17/Treg balance shifting to Th2 and Treg dominant, and trophoblast and immune cells expressing iDO and so on (**Figure 1**).

(1) Immune cells and IDO mechanism in maternal fetus immune tolerance

Cellular immunity is the central link of maternal immune tolerance to fetus. IDO is involved in maternal immune tolerance to fetus by cellular immunity. The expression level of IDO is low under normal conditions, and lipopolysaccharide and IFN-γ can induce the expression of IDO. From the early pregnancy, trophoblasts, NK, macrophages, DC and T cells at the maternal infant interface began to express IDO, and the amount of IDO gradually increased (53). IDO is a monomer enzyme of heme and the only limiting rate enzyme outside the liver that can catalyze tryptophan metabolism. IDO decomposes tryptophan along the kynurenine (KYN) pathway and produce a series of metabolites including quinolinic acid. Tryptophan is an essential amino acid to maintain cell activation and proliferation. IDO mainly suppresses cellular immunity through depletion of tryptophan (53). Firstly, iDO degrades L-tryptophan in local microenvironment, and limits T lymphocyte proliferation. Secondly, iDO produces KYN by metabolizing L-tryptophan (54). KYN is an endogenous ligand of Aromatic hydrocarbon receptor (AhR). KYN combines with AhR to cause immature CD4^+^ T cells to differentiate into inhibitory T cells. In addition, the combination of KYN and AhR can also induce the expression of IDO and further inhibit the immune response of T cells. Macrophages expressing IDO inhibit T cell function and promote the polarization of macrophages to M2 phenotype. IDO also inhibits NK cell activation by down regulating the surface receptors of NKp46 and NKG2D.

(2) HLA-G mainly expressing by trophoblast cells in maternal fetal immune tolerance (55)

Human trophoblast cells do not express MHC II molecule, HLA-A and HLA-B molecules of classical MHC I molecule, but express classical HLA-C and non classical HLA-E, HLA-F, and HLA-G to protect from cellular immune damage. HLA Ib molecules are the key to the generation and maintenance of maternal fetal immune tolerance. The research mainly focuses on the mechanism of immune escape induced by HLA-G. At present, HLA-C, E is also considered important. In the maternal-fetal interface, HLA class Ib molecules expressed in trophoblast cells were binded to inhibitory receptors of decidual immune cells (DICs), and then shifted Th1/Th2 balance toward Th2 bias. HLA-G as immune tolerance molecules is expressed in EVT of placenta and immune cells *in vivo*. There are two main mechanisms for HLA-G to induce immune tolerance. The first mechanism is that membrane-bound or soluble type of HLA-G can bind to the inhibitory receptors of immune cells (NK, T, B, monocytes and DC), which is a direct inhibition of immune cells. The second mechanism includes inducing tolerance of DC (56). HLA-G specifically inhibits T cell amplification and function in a dose related form (57, 58). The HLA-G antigen specifically combines to the ILT-4 receptor on the surface of DC, activates the downstream IL-6/STAT3 signal pathway, downregulates the expression of MHC-II and costimulatory molecules CD80/CD86, inhibits the maturation and differentiation of DCs, and induces the formation of tolerant DCs (56, 59). HLA-G also mediates the function of MDSC by ILT-4 receptor (58). HLA-C expressed by trophoblast cells can bind with KIRs of dNK cells and take part in trophoblast invasion into the uterine (60). HLA-G and HLA-E molecule expressed by EVTs can combine with the inhibitory receptor CD94/NKG2A of dNK cells, thereby inhibit dNK cell activation (61, 62). Other researchers found that HLA-G molecules could also promote the expression of HLA-E molecules, thereby indirectly reducing the toxicity of dNK cells (63).

**Figure 2:**
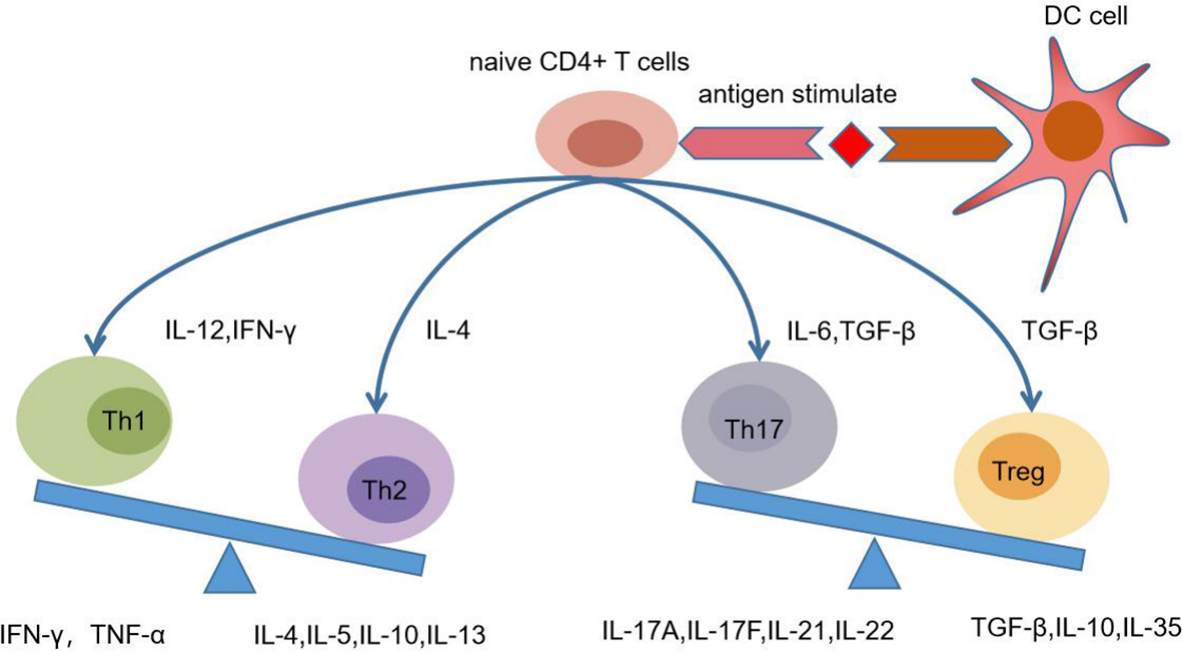
Naive CD4^+^ T cells differentiation in the pregnancy. In different microenvironment, naive CD4^+^T cells can differentiate into Th1, Th2, Th17, and Treg under the antigen stimulation which is presented by dendritic cells (DC). For example, IL12 and IFN-γ can induce the differentiation of Th1 cells, which secrete cytokines IFN-γ and TNF-ɑ. In the Th1/Th2 balance during pregnancy, Th2 is dominant. In the Th17/Treg balance, Treg was dominant.

(3) Th1/Th2/Th17 and Treg cell models in maternal fetal immune tolerance (64–66) (**Figure 2**).

The differentiation of CD4^+^ T cells is affected by many factors and cytokines. It is generally believed that Th cell precursor can differentiate into Th0 cells under the stimulation of antigen. In different microenvironments, Th0 cells can selectively differentiate into Th1, Th2, Th17 and Treg (**Figure 2**). It is traditionally believed that the Th1/Th2 balance at the maternal fetal interface during pregnancy tends to Th2 dominant (67–69). Th1 cells can release cytokines that cause local inflammation and activate MФS, NK cells and T lymphocytes. Th2 cells can release cytokines and promote humoral immune response. Th2 cells was once considered as the main T cell subgroup that assisted B cells to produce humoral immune response. The latest research suggests that follicular helper T cell (Tfh) belonging to CD4^+^ T is the main subgroup of helper B cells (70), and plays a regulatory role in B cell activation and Ig class conversion.

Treg/Th17 maybe play a much more important immune regulation role than Th1/Th2. At present, it has developed to Th1/Th2/Th17 and Treg cell models (71). Th17 cells have strong proinflammatory effect and can secrete IL-17A, IL-17F, IL-22, IL-21 and other cytokines. Th17 cells are differentiated by TGF-β, which can also induce regulatory T cells (iTreg) (72). IL-17 is the main effector of Th17 cells, which has proinflammatory effect which is in favor of the production of proinflammatory cytokines and chemokines (70, 73). The symptoms of RA usually improve during pregnancy, indicating that Th17 cells may decrease during pregnancy (74). The shift of Th17/Treg balance to Treg dominant may be favor to the outcome of pregnancy (75).

(4) Changes of pregnancy-associated hormone level in the maternal fetal immune tolerance

Hormonal factors are relative to maternal fetal immune tolerance including estrogen and progesterone and HCG. HCG can be detected from maternal serum on the first day after implantation of the fertilized eggs, reaching a peak at 8-10 weeks of gestation, and then dropping rapidly. Estrogen is produced by corpus luteum in early pregnancy, and is mainly synthesized by placental unit after 10 weeks of pregnancy. The concentration of estrogen gradually increases until the third trimester of pregnancy. Progesterone is produced by the corpus luteum of ovarian in the early pregnancy. After 8-10 weeks of pregnancy, placental syncytiotrophoblast cells begin to produce progesterone. The local concentration of the above hormones in the placenta is much higher than that in the whole body, which is mainly a regional effect and has little impact on all systems in the whole body.

During pregnancy, the hypothalamic neuropeptide corticotropin releasing hormone (CRH) mainly comes from endometrium and placenta. CRH induces the expression of Fas ligand (FasL) in DCs at the fetal maternal interface and increases apoptosis of activated T lymphocytes through Fas-FasL induction (76). High levels of adrenocortical hormone during pregnancy inhibit the immune system and directly inhibit NK cell activity. Cortisol secretion during pregnancy increased several times, and the active free cortisol was only 10% (77). Glucocorticoid (GC) can inhibit APC and Th1 cells which produce IFN-α, IFN-γ, TNF-α and IL-12, and promote Th2 cells which produce IL-4, IL-10 and IL-13 (78). The increase of GC level may lead to the maternal fetal local Th1/T2 to Th2 type, but it is not systemic inhibition (78).

Estrogen and progesterone levels increase during pregnancy. Research proved that progesterone not only increased the proportion of CD4^+^ CD25^+^ Treg cells and the expression of IL-10, but also enhanced its inhibitory function in mice (79). 17β-Estradiol (E2) can start immunosuppression through CD4^+^ CD25^+^ Treg cells in early pregnancy, reduce the production of Th1/Th17 inflammatory cytokines and up regulate the production of anti-inflammatory cell factors (80). Progesterone induces Tregs amplification, which is mediated by glucocorticoid receptors (81). Progesterone promotes Th2 cytokine production during normal pregnancy (82). In mice, progesterone directly or indirectly induced DC immune tolerance (83). Progesterone inhibits the activation and proliferation of lymphocytes (84). Progesterone can also inhibit the production of proinflammatory cytokine IL-17 (80). HCG can not only increase the number and activity of Treg, but also promote the migration of Tregs from peripheral blood to the maternal fetal interface (35, 85). In animal experiments, estradiol triggers expansion and activation of regulatory B cells. High level of HCG in pregnant women can induce Breg cells to expand, promote Breg to produce IL-10, and inhibit the proliferation of CD4^+^ T cells (86). During pregnancy, high levels of progesterone and estrogen promote humoral immunity by activating the T follicular helper cells (TFH)/B cell axis (87).

### Three hypotheses of immune tolerance in pregnancy

There are three hypotheses about the formation of immune tolerance in pregnancy as follows: Self-non-self models of immunity, danger model and Evolutionary non-self model. Self-non-self models of immunity, that is, if “non self” is recognized, antigen specific cytotoxic T lymphocytes (CTL) will be activated through a series of intermediate links. If it is “self”, antigen specific CTL will not be activated. Maternal fetal immune tolerance during pregnancy is achieved by limiting antigen specific T cell activity (88, 89). For the danger model, if there is no danger signal, the antigen expression from the father will not activate T cells (90). Dangerous signals are related to the activation of DCs or their costimulatory signals in the process of activating T cells. The danger model was validated in adverse pregnancy outcomes. “Evolutionary non-self” model: The presence of fetal antigen on the maternal fetal interface may not activate the immune system, but when infection occurs, the immune response mediated by PRR occurs to protect the maternal fuction (91). In short, the “Evolutionary non-self” model is more accurate in distinguishing between self and non self, and can accurately distinguish “microbial non self”, “changed self”, etc.

4) Most chronic HBV infected pregnant women in the “HBV immune tolerance period” before pregnancy

In China, most chronic HBV infections are transmitted by MTCT. After chronic infection, there are four periods: immune tolerance period, immune clearance period, immune control period and reactivation period. After acute HBV infection, CD4^+^ T cells secrete cytokines, activate CD8^+^ T cells, and kill HBV from infected hepatocytes through cell lysis and non lysis cell mode. HBV specific CD4^+^ T cells also stimulate B cells to produce antibodies and neutralize free viruses. This antiviral response is often unsuccessful in the immune tolerance period of chronic HBV infected patients, mainly manifested by the dysfunction or exhaustion of HBV specific CD8^+^ T cells (92). In chronic HBV infection, HBV specific CD8^+^ T mediated liver injury is caused firstly by antigen non-specific cells (such as NK cells). Age is an important factor in determining the immune tolerance period (93). It is generally believed that once age exceeds 30 or 40 years old, the immune tolerance is gradually broken (94). Most women of childbearing age (25-35 years old) are in the HBV immune tolerance period, with high HBsAg, HBeAg positive, and HBV DNA>10^8^IU/ml. During immune tolerance period, the immune cells will not directly or indirectly attack the liver cells, and the liver inflammation is very slight. The condition of chronic HBV infection women during pregnancy is relatively stable, with few HBV related progress or liver failure (95). In 2008, it was reported that in 35 pregnant women without antiviral treatment, abnormal liver function were 34.6% during pregnancy and 50% after delivery (4). There was no statistical difference in the rate of postpartum hepatitis in receiving AVT group in late pregnancy compared with not receiving AVT group (39.4% vs 38.7%, *P*=0.942) in chronic HBV infection pregnant women (96). During pregnancy, high levels of progesterone and estrogen and Glucocorticoid are favour to the Th2 and Treg dominant mode accompanied by the increase of corresponding cytokines. To sum up, chronic HBV infection pregnant women in immune tolerance period before pregnancy remain keep “immune tolerance to HBV” during pregnancy (97).

There are conflicting results about the quantitative changes of HBV DNA during pregnancy. This may be caused by the mix factors such as the natural stage of chronic HBV infection, HBeAg status and the level of HBV DNA before pregnancy. In the Liu J’study, HBeAg positive pregnant women group was enrolled with baseline HBV DNA >7.0 log_10_IU/mL, HBsAg >4.0 log_10_IU/mL and HBeAg >3.0 log_10_S/CO in the pregnancy (98). It has been found that in pregnant women without AVT in late pregnancy, no matter whether the ALT is normal or abnormal during pregnancy and postpartum, HBV DNA, HBsAg and HBeAg quantification have almost remained stable (98). However, some studies have found that HBV DNA had elevated in some patients during pregnancy. Chang CY found that about 9% of chronic HBV infection pregnant women with low baseline virus load had elevated HBV DNA levels during pregnancy (99). It was also found that there was an increasing trend on HBV DNA level during pregnancy, with average value of 0.4-1log_10_IU/ml (100). Analysis of causes: On the one hand, high levels of adrenocortical hormone during pregnancy may increase HBV replication (101). The increase of corticosteroid during pregnancy can activate glucocorticoid response elements in HBV gene, further enhance HBV replication and HBV gene expression. But the increase of estrogen level during pregnancy has been proved to reduce HBV replication in animal experiments (102). On the other hand, the predominance of Th2 in chronic HBV infection women during pregnancy is conducive to maintain immune tolerance to HBV *in vivo* (103).

## Maternal change on immune system and hormone after delivery

**Figure 3:**
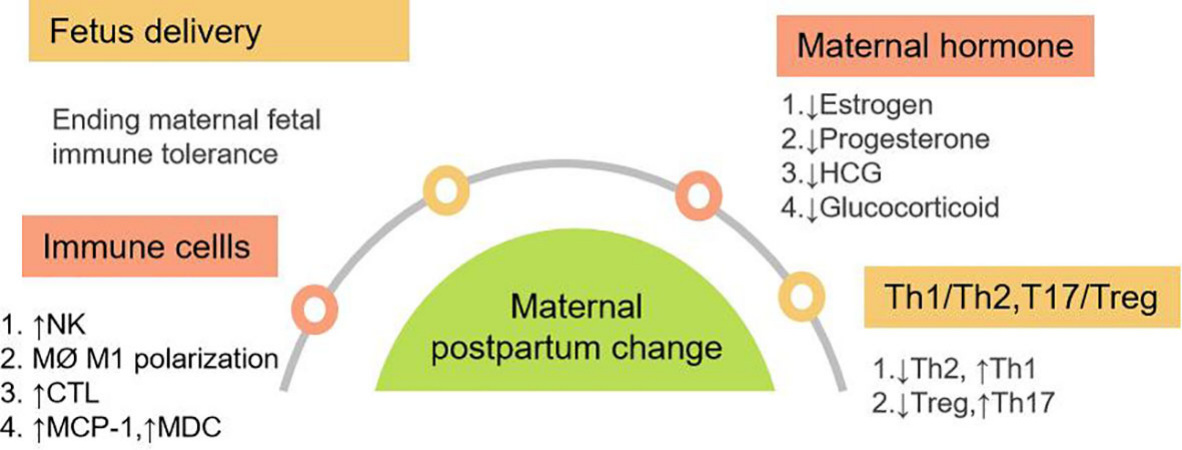
Maternal Postpartum changes in immune system and hormone. It mainly includes four aspects: Fetal delivery; changes of immune cells; changes in maternal hormone levels; changes of Th1, Th2, Th17 and Treg balance *in vivo*. Abbreviation: MCP-1: monocyte chemoattractant protein-1, MDC:macrophage derived chemokine.

After the fetus is delivered, maternal fetal immune tolerance is no longer required and maternal cell immunity gradually recovers (**Figure 3**). In the first trimester pregnancy, moderate inflammatory reaction is conducive to placenta implantation, and inflammation is gradually reduced in the second trimester pregnancy. In the third trimester of pregnancy, the uterine is again in an inflammatory state, so as to prepare for the delivery of the fetus and the face of birth canal infection.

### Changes of innate immunity and adaptive immunity after delivery.

Both innate and adaptive immunity were activated after delivery. NK cells gathered at the maternal fetal interface during pregnancy returns to the peripheral blood after delivery. The increase of NK cell activity in postpartum mothers was mainly caused by the change of single NK cell activity (104). The percentage and absolute count of NK cells after delivery increased temporarily (9). It was found that the frequency of NK cells increased from 6 weeks after delivery and held plateau at 6-24 weeks (105). Postpartum neutrophils increased. Postpartum monocyte chemoattractant protein-1 (MCP-1), also known as CCL2 and macrophage derived chemokine (MDC) levels increased after delivery (106). As CC subclass chemokines, MCP-1 and DC can cause chemotactic migration of dendritic cells, monocytes and T cells, especially Th1 cells. During labor, macrophages in the cervix rapidly aggregate, showing M1 proinflammatory phenotype, and participate in regulating cervical maturation and the beginning of labor by producing proinflammatory cytokines (15). In the mouse model, it was found that postpartum cervical macrophages obtained M2 phenotype, which may be related to postpartum tissue repair (107).

Postpartum adaptive immune adjusts accordingly. In not receiving AVT patients with chronic HBV infection, the increase of Th1 cytokines during perinatal period can be slight. Due to the lack of IDO and HLA-G, their inhibition on immune cells is relieved. Th and CTL increased from 1 to 4 months postpartum, while inhibitory T cells increased at 7 months postpartum (108). As an important component of adaptive immunity, B cells can produce specific antibodies, present antigens and regulate immunity. CD5^-^ and CD5^+^ B cells further decreased at 1 month postpartum, but CD5^+^ B cells significantly increased at 7-10 months postpartum (108). Plasma IL-6 and hsC-reactive protein (hsCRP) levels were higher in the early postpartum period. After delivery, IFN-γ, IL-2 and TNF-α were detected to be low level, and recovered 3-4 months later (109).

In a word, after the delivery of the fetus, maternal immunity presents Th1 and T17 dominant. The specific cellular immunity gradually increases in 3-4 months (109). Postpartum humoral immunity also increased after 6 months. The change of immune system lasts for 1 year after delivery.

### Estrogen, progesterone, HCG and cortisol levels after delivery

HCG reached its peak at 8-10 weeks of gestation, then decreased rapidly, and disappeared within 2 weeks after delivery. Estrogen reached its peak at the end of pregnancy and decreased rapidly after delivery. Progesterone decreased rapidly after delivery. Postpartum progesterone rebound to baseline levels before pregnancy and no longer promotes the production of Th2 cytokine (110). Maternal HCG, estrogen and progesterone levels will recover in one month and the decrease of estrogen, progesterone and HCG promote the transformation from Th2 and Treg dominant in pregnancy to Th1 and Th17 dominant after delivery.

In conclusion, there is no need for maternal fetal immune tolerance after delivery. In the background of uterine inflammation and disappearance of immune tolerance after delivery, the immunosuppression in innate and adaptive immune is reversed. Under the influence of various postpartum factors, there are shifts of Th1/Th2 to Th1 dominant and Th17/Treg to Th17 dominant type. The rapid recovery of hormones of estrogen, progesterone, HCG and cortisol levels aggravate the inflammatory process after delivery. (**Figure 3**)

## Postpartum hepatitis and maternal changes

**Figure 4:**
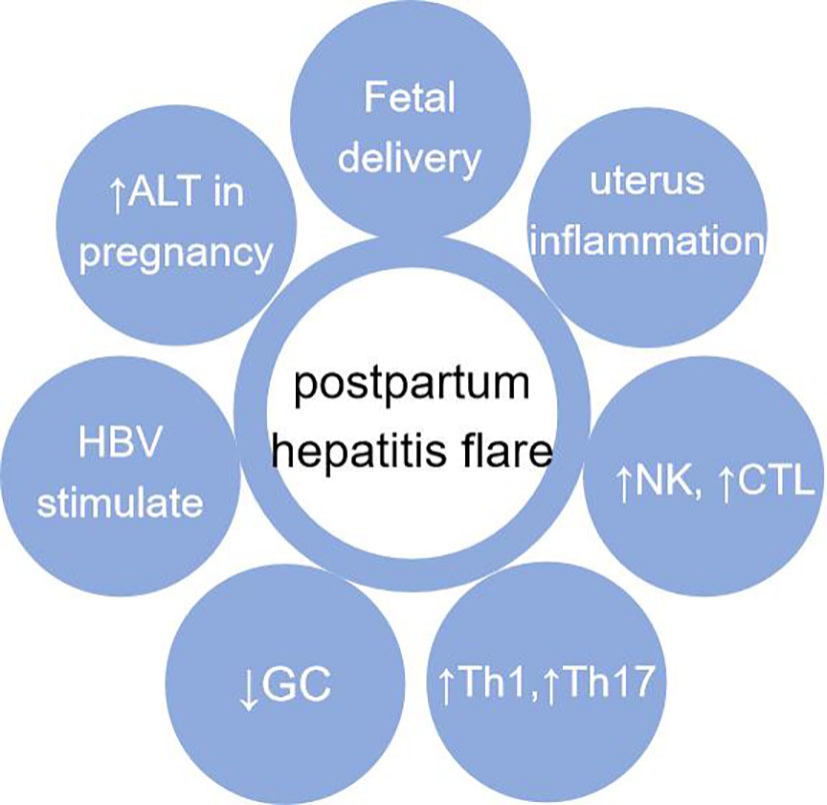
Causes of postpartum hepatitis. It mainly includes: fetal delivery (maternal fetal immune tolerance disappears);local inflammation of uterus after delivery; recovery of immune system after delivery; changes of Th1, Th2, Th17 and Treg balance after delivery; after delivery, GC decreases which is similar to “hormone withdrawal” reaction; HBV stimulation. Abbreviation: GC, Corticosteroid.

The causes of postpartum hepatitis are complex. Although immune tolerance to antigens during pregnancy mainly occurs at the maternal-fetal interface, it also affects the systemic immune system. With postpartum uterine inflammation, the body immunity is mainly Th1 and Th17 type. The change of postpartum hormone level does not seem to directly cause postpartum hepatitis, but it can make Th1 cells predominate. The rapid decrease of postpartum cortisol level is similar to the effect of stopping cortisol, which reduces the inhibition of liver inflammation and may aggravate the liver inflammatory reaction (111, 112). In the above condition, if stimulated by HBV DNA, postpartum hepatitis will flare (**Figure 4**).

### Postpartum hepatitis with innate and adaptive immunity

The changes of innate immunity and adaptive immunity after delivery mainly lead to postpartum hepatitis flare. The immune effect of pregnancy lasts about 1 year after delivery (108). After delivery, the immune tolerance to the fetus disappears rapidly. The number and function inhibition of innate immune cells often recovery earlier after delivery. There are many reasons for postpartum hepatitis, including proinflammatory factor release, the withdrawal of corticosteroids, especially the enhancement of HBV specific T cell response induced by viral HBV DNA, cytokines, etc. Postpartum hepatitis should also exclude various common causes of abnormal liver function, such as non-alcoholic fatty liver disease and drug-induced liver damage.

The largest number of innate immune cells in the liver is NK, followed by macrophages. After delivery, the delivery woman is in an inflammatory state caused by bacteria or other reasons. Postpartum inflammatory cytokines and chemokines may also activate innate immunity or adaptive immunity, causing hepatitis. The number and activity of NK cells in postpartum peripheral blood increased. Liver NK cells may cause liver inflammation through a non-antigen specific mechanism through (TRAIL/TRAIL R) mediated apoptosis, which can be initiated by cytokines such as IFN-α (113). Under the stimulation of inflammation, macrophages derived from Kupffer cells or monocytes in the liver are activated. Macrophages play the role of double swords. On the one hand, they are conducive to controlling infection, on the other hand, they can release chemokines, recruit more monocytes and lymphocytes to the liver, damage liver cells, and promote the occurrence of fibrosis.

In cellular immunity, CD8^+^ T plays a major role in immune cells proliferation and activation. It has been reported that the postpartum ALT and Th1 cytokines in untreated CHB patients increased slightly, and the serum HBsAg and HBV DNA content did not change (95). It is considered that in this study the increase of Th1 cytokines is not strong enough to cause immune suppression to HBV after postpartum (95). Compared with the group without postpartum hepatitis, the number of Treg in delivery woman with postpartum hepatitis was low, the ratio of CD4^+^ T cells expressing activated marker CD69 was significantly higher. In postpartum hepatitis mother, CD4^+^ T cells secreted more IFN-γ, IL-21, IL-2, TNF-α, and secreted more pro-inflammatory/anti-inflammatory cytokines (114). After delivery, CD8^+^ T and its subgroup effector memory T cell (Tem) and terminally differentiated effector memory T cells (TEMRA) in postpartum hepatitis flares group were significantly activated, and the expression levels of perforin and granzyme B increased (108, 115). In postpartum hepatitis flare group, activation of CD8^+^ T cells can produce TNF-α and IFN-α and induce postpartum hepatitis (114, 115). Finally, postpartum hepatitis may be accompanied by the decrease of HBV DNA, and HBeAg or HBsAg.

### Postpartum hepatitis and chronic HBV infection

#### Postpartum hepatitis and the natural course of chronic HBV infection

For the first pregnancy, most HBeAg-positive women of childbearing age with chronic HBV infection are in the HBV immune tolerance period, and a small part are in the immune control period. ALT and HBeAg status can help us distinguish immune tolerance status from immune clearance phase (116). Most pregnant woman are in the period of immune tolerance or immune control before pregnancy. At the early stage, it is believed that pregnancy and childbirth have little impact on the natural process of chronic HBV infection. Women with chronic HBV infection rarely develop severe HBV related liver disease during pregnancy, which may be due to the predominance of Th2 cells in women during pregnancy. Early research supported that the virology and serology of chronic HBV infection were no significant change in pregnancy and postpartum (98). The quantification of HBV DNA, HBsAg/HBeAg had no significant change from pregnancy to 12 months after delivery in patients with e antigen positive or e antigen negative chronic HBV infection. But now it seems that serological conversion of HBeAg can occur after delivery. As an immune tolerance factor, HBeAg is also a risk factor for postpartum hepatitis. Postpartum hepatitis in HBeAg positive women was 2.56 times higher than that in HBeAg negative delivery women (117). Postpartum immune reactivation can lead to increased ALT, and women with HBeAg positive with low titer can transit to the immune clearance period accompanied by the decline of HBsAg and HBeAg, and even HBeAg serological conversion after delivery (117). Postpartum HBeAg serum conversion rate ranged from 7-17% (117–119). Compared with non pregnant women, the HBeAg seroconversion rate of pregnant women with an average age of 30.7 ± 3.6 years increased from 2.2% to 14.3% (4). The clearance rate of HBeAg in HBeAg positive and NAVT delivery women was closely related to decrease in both HBeAg titer and/or HBV-DNA concentration in 1-2 months after delivery (118). Forty HBeAg positive pregnant women were studied in Taiwan, with an average age of 29.9 ± 5.3 years. During the follow-up period from the beginning of labor to 1 year, it was found that prepartum HBeAg titer<1:650 was associated with high HBeAg seroconversion rate, and there was no significant correlation with genotype B and genotype C (119). Observing the data of 214 HBeAg positive and NAVT delivery woman, HBsAg<1.0×10^4^IU/mL, and HBeAg<7.36×10^2^S/CO, HBV DNA level<1.0 ×10^7^ IU/mL at delivery were independent predictors of HBeAg seroconversion within 12 months postpartum (120). The presence of PC, BCP, and PC and BCP mutations during labor was associated with an increased likelihood of spontaneous postnatal HBeAg seroconversion (all P<0.05) (120). Other studies have similar findings. The NAVT HBeAg positive delivery woman were observed for 6.4 years. The baseline HBV DNA<3x10^7^IU/mL or HBeAg<770 S/CO in pregnancy was the predictive factor of HBeAg serological conversion (121).

#### Relationship between postpartum hepatitis and quantitative of HBsAg and HBV DNA in pregnant women with chronic HBV infection

Because of the interaction between HBV and immune response, postpartum hepatitis is more common in chronic HBV infection women. HBV DNA during delivery is also a predictor of postpartum hepatitis. There is 30% probability for postpartum hepatitis in pregnant women who can detect HBV DNA during delivery, and 20% probability in pregnant women who do not detect viremia in chronic HBV infected patients (2). The study found that hepatitis during pregnancy was 4.6% (2/44) and 7.1% (5/68) respectively for HBV DNA<2000 IU/mL and HBV DNA>2000 IU/mL, and hepatitis after delivery was 7.4% (2/28) and 13.0% (3/23) respectively (99). Some studies believe that the cutoff value of postpartum hepatitis is HBV DNA ≥ 5 log_10_IU/mL at the delivery of untreated chronic HBV infected pregnant women with positive predictive value is 14.4% and the negative predictive value is 98.2% (2). It is speculated that the postpartum hepatitis flare can only be triggered when the HBV DNA load reaches a certain level. Among HBeAg negative pregnant women, about 30% of pregnant women have postpartum hepatitis, which may be related to reactivation in the immune control period. Age is related to HBV immune tolerance. It is believed that some 30 years old HBeAg positive women are in the transition stage from immune tolerance to immune clearance. The study found that once postpartum hepatitis occured, the quantitative level of HBsAg in delivery women would decrease significantly at 6-8 weeks or 15-18 weeks (114). HBsAg<100 IU/ml was the predictive factor of HBsAg seroconversion after delivery (121).

#### High risk factors of postpartum hepatitis

Early articles suggested that HBV DNA, ALT level, HBeAg status or any other characteristics of pregnant women could not predict postpartum hepatitis during pregnancy (51). However, in recent studies, the pregnant women were further differentiated according to the natural period of chronic HBV infection and HBeAg positive, and it was found that there were characteristic indicators to predict the onset of postpartum hepatitis (**Table 1**). In addition to the mentioned HBeAg positive and <700S/CO, HBV DNA>5Log_10_IU/ml at delivery is a predictor of postpartum hepatitis. Postpartum hepatitis can occur in chronic HBV infected people receiving AVT or NAVT (120–123). The increase rate of hepatitis during pregnancy and after delivery was 14% and 16%, respectively (5). ALT at delivery is elevated, which is also a high-risk predictor of postpartum hepatitis (2). In 241 pregnant women, 33.6% of the women had elevated serum ALT during pregnancy. Multivariate analysis showed that elevated ALT during pregnancy was associated with postpartum hepatitis after telbivudine withdrawal (124). The cutoff value of predicting postpartum hepatitis in pregnant women was 29 years old, the prenatal ALT was greater than 14.8 U/L, and the postnatal HBeAg level was less than 3.1 log_10_ S/CO (125). Studies on pregnant women showed that ALT at 32 weeks of gestation and HBcAb were risk factors of postpartum hepatitis (126). HBeAg positivity and gestational diabetes were associated with postpartum hepatitis (123). When oral AVT is used in late pregnancy and stopped after delivery, the prediction model of postpartum hepatitis includes the decrease of HBsAg and HBV DNA from baseline to gestation 32 weeks or delivery, respectively (127).

**Table 1:** The characteristics of the references on risk factors of postpartum hepatitis flare.

author	Year	(N)	regions	Age(years)^a^	standard^b^ (U/L)	Rate	Risk factors	AVT (N)^c^
Giles M (117)	2015	126	75%Asian	31.45 ± 0.43	>2ULN	25%	HBeAg positive at baseline Is 2.56 times	7
Liu J (124)	2017	241	Chinese	27 (20–40)	>1ULN	33.6%	ALT elevation at pregnancy	241
Kushner T (5)	2018	310	African and Asian	30 (25-33)	>2ULN	16%	HBeAg positive	21
Yi We (2)	2018	3367	Chinese	29.15 ± 4.16	>5ULN	3.50%	elevated ALT, HBV DNA ≥5 log_10_IU/mL at delivery	0
Li L (123)	2020	317	Chinese	29.3 ± 4.2	>1.5ULN	16.7%	HBeAg positiv, gestational diabetes	138
Lu J (125)	2021	153	Chinese	28.8 ± 3.88	>2ULN	36.60%	age<29 years, antepartum ALT>14.8U/L, postpartum HBeAg<3.1 logCOI	153
Quan M (126)	2022	444	Chinese	29 (26, 31)	>1ULN	52.93%	ALT at 32 weeks of pregnancy, HBcAb at intrapartum	444
	2022	170	Chinese	28 (26, 30)	>1ULN	70.59%	–	0

^a^Data are presented as mean ± Standard Deviation or median, ^b^The standards for postpartum hepatitis, ^c^The number of chronic HBV infection pateints receiving AVT in late pregnancy. ULN, upper limit of normal range.

At first, scholars questioned the correctness of AVT in late pregnancy. According to previous research reports, 62% of women will have postpartum hepatitis after stopping antiviral treatment after delivery, which is significantly higher than that of women who do not receive antiviral treatment (36% vs 62%) (51). Ayres et al. reported that although short-term lamivudine treatment during pregnancy moderately reduced maternal viremia, HBV rtM204I/V and rtA181T mutations were detected (128). With the emergence of powerful and highly resistant barrier drugs, guidelines recommend antiviral treatment for pregnant women in late pregnancy with high viral load of hepatitis B virus infection.

Whether stopping AVT after delivery is a risk factor for postpartum hepatitis in chronic HBV infected women who have received oral nucleoside/nucleotides analogs in late pregnancy? It is believed that even if prophylaxis MTCT treatment continues until 12 weeks postpartum, postnatal hepatitis will still occur during this period. It is believed that postpartum hepatitis is generally not serious and reversible if preventive AVT is stopped after delivery (124). Postpartum hepatitis occurred in 25% of women with elevated ALT during pregnancy after discontinuation of telbivudine (124). In immune related studies, lower IFN-α during pregnancy and higher IFN-α after delivery may be related to postpartum hepatitis (125). In the population receiving antiviral treatment, the number of Treg is relatively reduced in chronic HBV infected women with postpartum hepatitis, the production of pro-inflammatory factors IFN-γ, IL-2 and TNF-α is more, and the production of anti-inflammatory cytokines (IL-10) is less (114).

(4) Postpartum hepatitis and treatment to prevent MTCT

AVT in the third trimester of pregnancy can reduce the incidence of MTCT (129–132). After delivery, the immune reactivation in chronic HBV infection women is the basis of hepatitis flare (106). In addition, many factors are involved in the occurrence of postpartum hepatitis. During AVT, there is a negative correlation between the enhancement of Th1/Th2 immunity and the decrease of HBV DNA load (133). Oral AVT reduces HBV DNA load which may further promote to the recovery of HBV specific T cell function. Treatment of prevention MTCT can obviously increase the frequency of postpartum CD83^+^ pDCs and CD86^+^ pDCs (134). The increase in the frequency of differentiated mature DC means that the immune presentation function is enhanced, which is conducive to activating specific cellular immunity. HBV specific immune enhancement participates the damage of HBV infected liver cells, on the other hand, it is conducive to the HBeAg seroconversion. The incidence of postpartum hepatitis after withdrawal of AVT after delivery ranged from 5% to 62%, which may be related to hepatitis postpartum criteria, the frequency of ALT monitoring, and the withdrawal time of AVT. Postpartum immune effect was maintained for 1 year (108). Professor Xie Yao’s team conducted a prospective study on pregnant women with HBeAg positive chronic HBV infection, including 96 cases of withdrawal the drug immediately postpartum, and 37 cases of were delayed withdrawal to six weeks postpartum (135). The conclusion of the study suggests that withdrawal of antiviral drugs immediately or six weeks postpartum does not affect the rate of postpartum hepatitis, but may delay postpartum hepatitis until twelve weeks postpartum (135). AVT in late pregnancy prolonged to 12 weeks postpartum did not decrease the occurance rate of hepatitis after delivery compared with immediate withdrawal postpartum. In pregnant women who with elevated ALT prolonged AVT to 52 weeks postpartum, HBeAg serum conversion rate increased significantly, and HBsAg titer decreased significantly (124).

Postpartum anti-HBV treatment is often a good time for achieving the seroclearance of HBeAg or HBsAg. In the chronic HBV infected women who were in immune tolerance period before pregnancy, some people would enter the immune clearance period after delivery. Previous studies have shown that in the pursuit of HBeAg seroconversion or clinical cure, a more effective treatment is the interferon-based treatment. If there is no contraindication of interferon, oral nucleoside/nucleotides analogs combined with pegylated-interferon can often achieve higher HBeAg seroclearance or HBsAg seroclearance. A research was conducted in pregnant women with ALT <1ULN, HBeAg positive and HBV DNA>6log_10_IU/ml who had received oral AVT in the late pregnancy. If the postpartum women showed the characteristics of ALT>2ULN with HBeAg decreased by more than 20% or HBV DNA decreased by more than 2log_10_IU/ml compared with baseline of late pregnancy, they were included in the study group which were given pegylated-interferon combined with Adefovir, while the control group stopped taking AVT orally in 12 weeks after delivery. In the study group, 56.7% (17/30) patients achieved HBeAg seroclearance and 26.7% (8/30) patients achieved HBsAg seroclearance. No one in the control group achieved the seroclearance of HBeAg or HBsAg (136).

In conclusion, during pregnancy, maternal fetal immune tolerance makes immunosuppression in specific immunity and non-specific immunity. The elevation of pregnancy related hormones will also affect immunity. The condition of pregnant women with chronic HBV infection seems to be relatively stable during pregnancy. Postpartum human immune system reverses the trend of maternal fetal immune tolerance and were dominated by uterine inflammation. NK’s non-specific immunity is enhanced, Th1 cells and corresponding cytokines are increased, DC’ antigen presenting function is enhanced. The content of serum cortisol dropped rapidly after delivery, which was equivalent to hormone withdrawal reaction. In delivery women with chronic HBV infection, when HBV DNA can be detected, it can often induce or aggravate the hepatitis flare. There are several indicators to predict postpartum hepatitis, including HBeAg positive and<700 S/CO, childbearing age and<29 years old, and HBV DNA>3-5Log_10_ IU/ml. When delaying the time for withdrawal AVT to 6-12 weeks postpartum, postpartum hepatitis rate cannot decrease. For pregnant women with elevated ALT during pregnancy, the HBeAg serum conversion rate can be increased by prolonging the AVT to more than 48 weeks if HBeAg tends to decline from the baseline in the pregnant or delivery. If the postpartum biochemical, virological or other indicators indicate that the delivery women have entered the immune clearance period, it is recommended to consider anti-HBV treatment. If there is no interferon contraindication, the interferon-based treatmment is more conducive to HBeAg or HBsAg seroclearance after delivery. In rare cases postpartum hepatitis flare would be severe, and oral AVT is recommended.

## Author contributions

ML, YLu, YJ and YX contributed to study concept and design. TJ, YY, WD, HL, SW, RL, MC, SWu, YG, HH, GS, XC, LH, LY, XB and YLin collected and sorted out literatures. LZ wrote the fifirst draft and drew pictures. TJ, YY, WD, HL and SW edited the English version. ML, YLu, YJ and YX modify the version to be submitted. All authors contributed to the article and approved the submitted version.
